# Cost-Effectiveness of Pre-Referral Antimalarial, Antibacterial, and Combined Rectal Formulations for Severe Febrile Illness

**DOI:** 10.1371/journal.pone.0014446

**Published:** 2010-12-29

**Authors:** James Buchanan, Borislava Mihaylova, Alastair Gray, Nicholas White

**Affiliations:** 1 Department of Public Health, Health Economics Research Centre, University of Oxford, Oxford, United Kingdom; 2 Faculty of Tropical Medicine, Mahidol University and Centre for Tropical Medicine, Churchill Hospital, Oxford, United Kingdom; Menzies School of Health Research, Australia

## Abstract

**Background:**

Malaria and bacterial infections account for most infectious disease deaths in developing countries. Prompt treatment saves lives, but rapid deterioration often prevents the use of oral therapies; delays in reaching health facilities providing parenteral interventions are common. Rapidly and reliably absorbed antimalarial/antibacterial rectal formulations used in the community could prevent deaths and disabilities. Rectal antimalarial treatments are currently available; rectal antibacterial treatments are yet to be developed. Assessment of the likely cost-effectiveness of these interventions will inform research priorities and implementation.

**Methods and Findings:**

The burden of malaria and bacterial infections worldwide and in Sub-Saharan and Southern Africa (SSA) and South and South-East Asia (SEA) was summarised using published data. The additional healthcare costs (US$) per death and per Disability Adjusted Life Year (DALY) avoided following pre-referral treatment of severe febrile illness with rectal antimalarials, antibacterials or combined antimalarial/antibacterials in populations at malaria risk in SSA/SEA were assessed. 46 million severe malaria and bacterial infections and 5 million deaths occur worldwide each year, mostly in SSA/SEA. At annual delivery costs of $0.02/capita and 100% coverage, rectal antimalarials ($2 per dose) would avert 240,000 deaths in SSA and 7,000 deaths in SEA at $5 and $177 per DALY avoided, respectively; rectal antibacterials ($2 per dose) would avert 130,000 deaths in SSA and 27,000 deaths in SEA at $19 and $97 per DALY avoided, respectively. Combined rectal formulations ($2.50 per dose) would avert 370,000 deaths in SSA and 33,000 deaths in SEA at $8 and $79 per DALY avoided, respectively, and are a cost-effective alternative to rectal antimalarials or antibacterials alone.

**Conclusions:**

Antimalarial, antibacterial and combined rectal formulations are likely to be cost-effective interventions for severe febrile illness in the community. Attention should focus on developing effective rectal antibacterials and ensuring that these lifesaving treatments are used in a cost-effective manner.

## Introduction

Infectious diseases are a leading cause of death in developing countries. Malaria and bacterial infections account for the majority of these deaths [1]. Prompt treatment of severe malaria or sepsis could save lives but clinical deterioration is often rapid, leading to inability to swallow medicines. The majority of deaths from febrile illness occur in children in or near home, before these patients can reach a facility where parenteral treatments can be provided [2]. Malaria and bacterial infections have overlapping symptoms [2] and often co-occur with infections such as septicaemia, relatively common in children with severe malaria [3], [4]. Malaria is also common in children hospitalised with severe pneumonia in malaria endemic regions [5]. Distinguishing between malaria, septicaemia and pneumonia is clinically difficult, particularly in young children. Consequently, as a pragmatic compromise, the Integrated Management of Childhood Illnesses (IMCI) strategy developed by WHO and UNICEF recommends that severely ill febrile children in malaria endemic regions are treated with parenteral antibiotics and antimalarials [6], [7].

Rectal drugs can be given safely to severe febrile patients, offering the prospect of providing potentially life-saving pre-referral (i.e. prior to referral to a healthcare facility) treatment to those who are seriously ill and are unable to take oral medications reliably. A large randomised trial of pre-referral community use of rectal artesunate (Gomes *et al*. (2009)) [2] showed that in 12,068 malaria patients unable to take oral treatment in Ghana, Tanzania and Bangladesh, mortality was halved in those who had not reached hospital within six hours of rectal artesunate administration.

This paper builds on that finding and considers the economic case for the development and use of antimalarial, antibacterial and combined antimalarial/antibacterial rectal interventions for severe malaria and severe bacterial disease in the community. Rectal formulations of antimalarial treatments have already been developed and are recommended for community use [7], [8], although not yet widely deployed. Rectal formulations of antibacterial treatment alone or in combination with an antimalarial are proposed to be developed to target a variety of bacterial infections. Lower respiratory infections (LRI - mostly pneumonia [9]) represent the group of bacterial infections with the largest reported global burden of disease and are the target bacterial disease of the hypothetical rectal antibacterial component studied here [1]. There are no significant drug interactions between parenteral antimalarials and antibiotics [7] so a combined approach is likely to be safe.

The objectives of this paper are to summarise the incidence and mortality of severe malaria and severe target bacterial disease both worldwide and in key regions and to estimate the likely cost-effectiveness of antimalarial, antibacterial and combined antimalarial/antibacterial rectal treatments.

## Methods

### Burden of disease

The burden of malaria and the target bacterial disease was summarised using published data both globally and for two specific regions: Sub-Saharan and Southern Africa (SSA - all countries in Africa except the non-malarious countries in Northern Africa) and South and South-East Asia (SEA – the malarious countries from the World Health Organisation (WHO) South-East Asia (SEARO) and Western Pacific (WPRO) regions). The presence of malaria in a country was informed by the 2008 World Malaria Report (WMR) [10]. Supplementary **Table S1** lists the countries forming the regional groupings. Data are reported for the under-five (excluding neonates) and five years and over age groups. Incidence rates are calculated using population data for 2006 [11], [12].

#### Malaria

The primary source of malaria incidence data was the 2008 WMR which reports data for ‘fever with parasites’, encompassing all vectors, for 2006 [10]. Severe malaria is mostly caused by Plasmodium falciparum [13]. No data sources for the burden of severe malaria were identified and, therefore, expert opinion estimates of the percentage of falciparum malaria incidence in SSA that was severe (5% of all cases in under-fives and 1% in the remaining population [14], [15], [16], [17]) and the percentage of falciparum malaria incidence elsewhere that was severe (2% across all ages) were employed. The proportion of malaria incidence attributed to falciparum infection at the regional level is based on the 2008 WHO GBD study [1]. Malaria mortality data were extracted from the WMR [10].

#### Target bacterial disease

No single source provided the data required to estimate the burden of the target bacterial disease. Pneumonia incidence amongst under-fives was based on a 2006 study of the epidemiology of childhood pneumonia in developing countries [18], [19] that reports incidence of ‘clinical pneumonia’: a definition consistent with WHO Case Management Guidelines [20] and IMCI Guidelines [8]. LRI incidence for all ages at the regional level was informed by the 2008 WHO GBD study [1]. These regional estimates were recalculated to include malarious countries only.

The proportion of pneumonia cases in under-fives that develop into severe pneumonia as defined in WHO treatment guidelines [18], [20] (8.6%) is based on Rudan *et al*. [18]. No equivalent estimate for those aged five years and over was identified; hence the same proportion is applied in this population. Our estimates of target bacterial disease mortality are based on total LRI mortality for 2004 reported in the 2008 WHO GBD study [1] and pneumonia mortality in under-fives reported in a 2004 UNICEF study [21].

### Cost-effectiveness

A decision model of the management of severe febrile illness was developed to evaluate costs, effects and cost-effectiveness of rectal treatments for people with severe febrile illness in populations at risk of malaria in SSA and SEA. While the whole SSA region is at risk of malaria, SEA is characterised by a mixed malaria risk profile, with areas of stable, unstable and no malaria risk. A recent study suggested that 45% of the SEA population is at risk of malaria [22]. We therefore assume that 45% of the target severe bacterial disease in SEA occurs in this population and the cost-effectiveness results we present are specific to this population.

**Figure 1:**
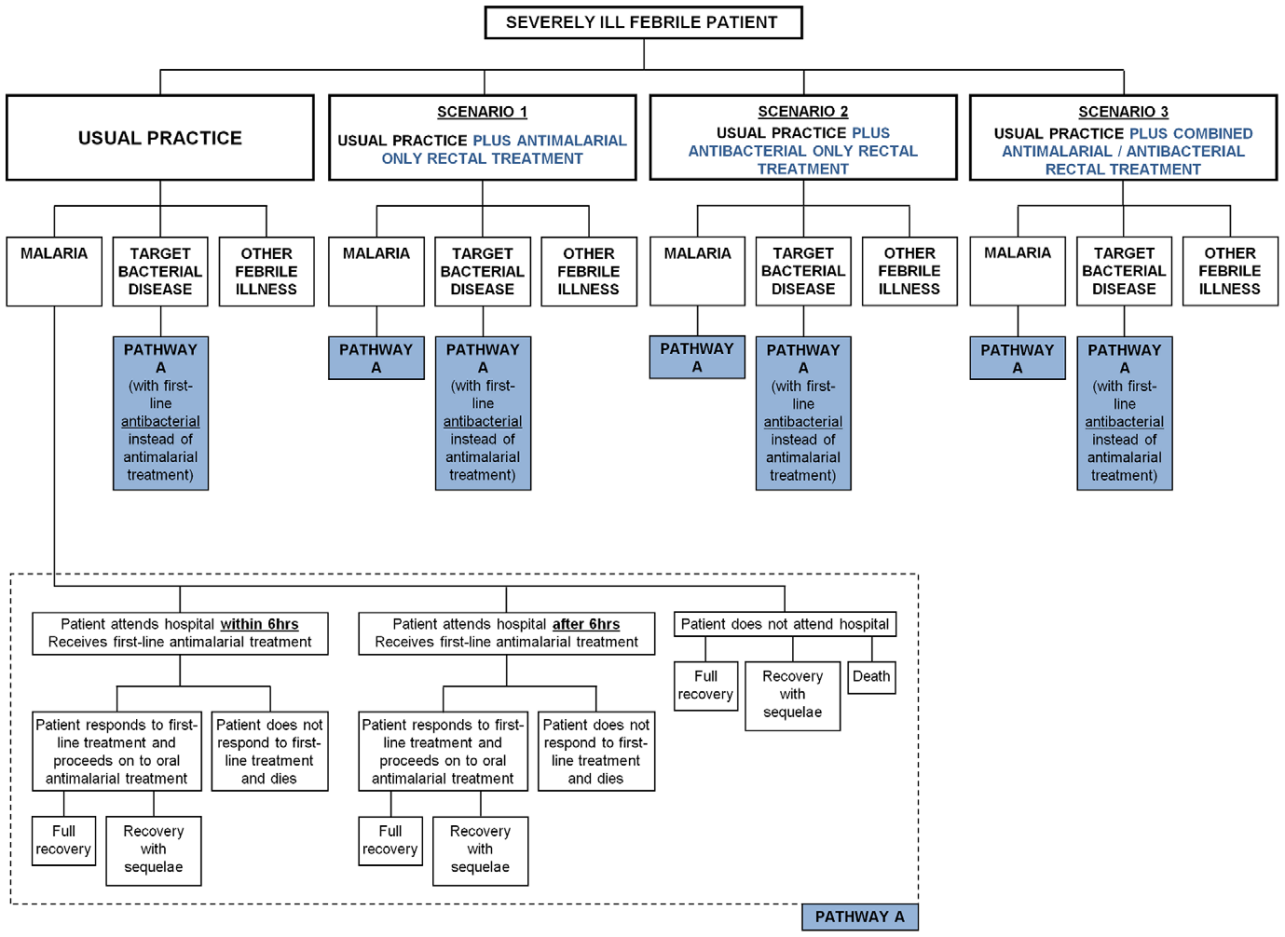
Schematic of the decision model of the management of severely ill febrile patients. Figure 1 illustrates the typical disease management of severe febrile patients in populations at risk of malaria in SSA and SEA, for each of the four scenarios of interest: usual practice; usual practice with antimalarial rectal treatment; usual practice with antibacterial treatment, and usual practice with combined antimalarial/antibacterial rectal treatment. ‘Hospital’ refers to a medical establishment able to provide parenteral and supportive treatment for a severe febrile patient. Pathway A  =  access to treatment for severe malaria or target bacterial disease. Usual practice refers to a situation where no rectal treatments for severe febrile illness are widely used. **Tables 1** and **2** contain details of all the parameters used within the model, for each region, including access to care rates, treatment effects and burden of disease.

The model evaluates the population health effects and costs of the current usual treatment practice, with no widespread use of pre-referral rectal treatment for malaria, as well as of three further health policies: (1) rectal antimalarial added to usual practice; (2) rectal antibacterial added to usual practice, and (3) a combined antimalarial/antibacterial rectal formulation added to usual practice (**Figure 1**). All model parameters are detailed in **Table 1**. In the model, patients with severe malaria or severe bacterial disease either attend a medical facility with the capacity to deliver parenteral treatment within 6 hours (from timing of rectal intervention administration), attend such care after 6 hours, or do not attend such care. These access-to-treatment categories reflect the treatment effects of rectal artesunate reported in Gomes *et al*. [2]. Access rates achieved in that study were high due to incentives provided within the study, and are unlikely to reflect usual practice; hence lower access rates are used in the decision model (**Table 1**, see also supplementary material). In the base case analysis the proportion of severe febrile cases who access health care is assumed to be unaffected by the use of rectal treatment.

**Table 1:** Parameters in the cost-effectiveness decision model for severe febrile illness.

Parameter	Base case value	Values for sensitivity and scenario analysis	Data sources for base case/sensitivity and scenario analysis
**Percent of cases that – access hospital or die within 6 hours/access hospital after 6 hours/never access hospital**			
SSA (Values for sensitivity analysis: lower access - higher access)	40%/40%/20%	15%/55%/30% -55%/32%/13%^1^	Asmp/Asmp/[2]
SEA (Values for sensitivity analysis: lower access – higher access)	80%/13%/7%	40%/40%/20% -93%/5%/2%^1^	Asmp/Asmp/[2]
**Disease and treatment related parameters – severe malaria**			
% of incidence that is severe disease (SSA) – under-fives/five years and over	5%/1%	2.5–7.5%/0.5–1.5%^2^	[14], [15], [16], [17]
% of falciparum malaria incidence that is severe disease (SEA) – all ages	2%	1–3%	Asmp [28]
Untreated case fatality rate – under-fives/five years and over	30%/50%	15–45%/25–75%	[23]
First-line treatment failure rate – under-fives/five years and over	6%/26%	3%/13%^3^	[27], [28]/[2]
Neurological sequelae incidence rate – all ages	5%	2.5–7.5%^2^	Asmp [2], [26], [31], [32]
**Disease and treatment related parameters – severe bacterial disease**			
% of all incidence that is severe disease - all ages	8.6%	4.3–12.9%^2^	[18]
Untreated case fatality rate – all ages	16%	8–24%	[18], [24]
First-line treatment failure rate – under-fives/five years and over	5.8%/9.8%	-	[29], [30]
Neurological sequelae incidence rate – all ages	0%	-	Asmp
**Rectal treatment effects – severe malaria**			
Reduction in mortality for patients who are alive but not in hospital within 6 hours of rectal treatment administration (RR)	51%	23–68%^4^	[2]/[2]
Reduction in neurological sequelae in all patients (RR)	42%	0–85%^5^	Midpoint [2], [33]/[2], [33]
Reduction in the untreated case fatality rate (RR)	20%	10–30%^2^	Asmp
**Rectal treatment effects – severe bacterial disease**			
Reduction in mortality for patients who are alive but not in hospital within 6 hours of rectal treatment administration	20%	10–30%^2^	Asmp
Reduction in the untreated case fatality rate	10%	5–15%^2^	Asmp
**Percentage of all patients treated with rectal formulations who do not have the target disease(s) and will not benefit from treatment**			
Antimalarial only/Antibacterial only/Combined	26%/10%/10%	-	[2]/Asmp/Asmp
**Costs (US dollars, 2005) – under-fives/five years and over**			
First-line parenteral antimalarial treatment – quinine^6^	$2.22/$9.68	-	[10], [35]
First-line parenteral antimalarial treatment – artemether^6^	$3.22/$14.05	-	[10], [35]
Oral antimalarial treatment – chloroquine and primaquine^7^	$0.32/$1.39	-	[10], [35]
Oral antimalarial treatment – artesunate and amodiaquine^7^	$0.27/$0.80	-	[10], [35]
Oral antimalarial treatment – artemether-lumefantrine^7^	$0.93/$2.79	-	[10], [35]
First-line antibiotic treatment – benzylpenicillin	$0.31/$2.06	-	[36]
Oral antibiotic treatment – amoxicillin	$0.22/$0.84	-	[36]
Antimalarial only rectal formulation - all ages	$2.00	$1.00–3.00^2^	Asmp
Combined rectal formulation - all ages	$2.50	$1.25–3.75^2^	Asmp
Antibacterial only rectal formulation - all ages	$2.00	$1.00–3.00^2^	Asmp
Rapid diagnostic test for malaria	$0.83	-	[35]
Cost per inpatient day at secondary level hospital in SSA	$25.17	$12.58–37.75^2^	[38]
Cost per inpatient day at secondary level hospital in SEA	$25.80	$12.90–38.70^2^	[38]
**Other parameters**			
Average length of stay (days) for patients who survive/survive with neurological sequelae/die	4.5/10/2	-	[14]/[39]/[14]
Life expectancy conditional on survival	Region-specific life tables 8	Japanese life tables 8	[44] [58]
Discount rate for future life years	3%	-	[1]
Disability weight for malaria patients with long-term neurological sequelae	0.471	-	[1]

RR = risk ratio; Asmp = Assumption; SSA-Sub Saharan and Southern Africa, SEA-South and South-East Asia;

1 Rates of access reported in Gomes *et al*. [2];

2 Parameter values varied by 50% above and below the base case value;

3 Parameter values varied by 50% below the base case value only, to reflect lower treatment failure rates in Gomes *et al*. [2];

4 95% confidence interval reported in Gomes *et al*. [2];

5 Parameter values varied between estimates reported in the two sources;

6 Artemether is used alongside quinine in SEA, hence the cost of first-line parenteral antimalarial treatment in this region was assumed to be an average of the cost of quinine treatment and artemether treatment [10];

7 National policies for treatment of uncomplicated falciparum malaria vary by country, Average costs were calculated for each region based on region-wide antimalarial drug policy as reported in the 2008 WMR [10];

8 Region-specific life tables were used to estimate life expectancy conditional on survival. Japanese life tables were used within a sensitivity analysis.

**Table 2:** Annual burden of malaria and target bacterial disease.

Malaria	Population	World	SSA^2^	SEA^2^	[Source] Data year
Incidence, thousands (*Episodes per person per year*)	Under five years	121,495 (*0.194*)	117,774 (*0.926*)	2,148 (*0.008*)	[10] 2006
	Five years and over	125,077 (*0.021*)	100,213 (*0.158*)	21,351 (*0.007*)	[10] 2006
	**TOTAL**	**246,572 (** ***0.037*** **)**	**217,988 (** ***0.286*** **)**	**23,499 (** ***0.007*** **)**	[10] 2006
Severe cases, thousands (*Episodes per person per year*)^1^	Under five years	5,930 (*0.009*)	5,889 (*0.046*)	25 (*0.0001*)	[10], [14] 2006
	Five years and over	1,280 (*0.0002*)	1,002 (*0.002*)	245 (*0.0001*)	[10], [14] 2006
	**TOTAL**	**7,211 (** ***0.001*** **)**	**6,891 (** ***0.009*** **)**	**269 (** ***0.0001*** **)**	[10], [14] 2006
Mortality, thousands (*Deaths per 1,000 incident cases*)	Under five years	751 (*6.185*)	736 (*6.245*)	14 (*6.587*)	[10] 2006
	Five years and over	130 (*1.037*)	101 (*1.007*)	26 (*1.199*)	[10] 2006
	**TOTAL**	**881 (** ***3.574*** **)**	**837 (** ***3.837*** **)**	**40 (** ***1.691*** **)**	[10] 2006
**Target bacterial disease**					
Incidence, thousands (*Episodes per person per year*)	Under five years	155,686 (*0.248*)	37,006 (*0.291*)	89,681 (*0.325*)	[19] 2006
	Five years and over	291,128 (*0.049*)	95,825 (*0.151*)	103,018 (*0.034*)	Residual
	**TOTAL**	**446,814 (** ***0.068*** **)**	**132,831 (** ***0.174*** **)**	**192,700 (** ***0.059*** **)**	Calculated based on [1], [19] 2004, 2006
Severe cases, thousands (*Episodes per person per year*)	Under five years	13,389 (*0.021*)	3,183 (*0.025*)	7,713 (*0.028*)	[19] 2006
	Five years and over	25,037 (*0.004*)	8,241 (*0.013*)	8,860 (*0.003*)	Residual
	**TOTAL**	**38,426 (** ***0.006*** **)**	**11,423 (** ***0.015*** **)**	**16,572 (** ***0.005*** **)**	Calculated based on [1], [19] 2004, 2006
Mortality, thousands (*Deaths per 1,000 incident cases*)	Under five years	2,044 (*13.129*)	1,047 (*28.292*)	627 (*6.991*)	[21] 2004
	Five years and over	2,133 (*7.325*)	407 (*4.243*)	1,113 (*10.799*)	Residual
	**TOTAL**	**4,177 (** ***9.348*** **)**	**1,454 (** ***10.943*** **)**	**1,740 (** ***9.027*** **)**	[1] 2004
**Malaria and target bacterial disease**					
Incidence, thousands (*Episodes per person per year*)	Under five years	277,181 (*0.442*)	154,781 (*1.217*)	91,830 (*0.333*)	Calculated
	Five years and over	416,205 (*0.070*)	196,038 (*0.308*)	124,369 (*0.042*)	Calculated
	**TOTAL**	**693,387 (** ***0.105*** **)**	**350,819 (** ***0.460*** **)**	**216,199 (** ***0.066*** **)**	Calculated
Severe cases, thousands (*Episodes per person per year*)	Under five years	19,319 (*0.031*)	9,071 (*0.071*)	7,737 (*0.028*)	Calculated
	Five years and over	26,318 (*0.004*)	9,243 (*0.015*)	9,104 (*0.003*)	Calculated
	**TOTAL**	**45,637 (** ***0.007*** **)**	**18,314 (** ***0.024*** **)**	**16,842 (** ***0.005*** **)**	Calculated
Mortality, thousands (*Deaths per 1,000 incident cases*)	Under five years	2,795 (*10.085*)	1,783 (*11.517*)	641 (*6.982*)	Calculated
	Five years and over	2,262 (*5.436*)	508 (*2.589*)	1,138 (*9.151*)	Calculated
	**TOTAL**	**5,058 (** ***7.294*** **)**	**2,290 (** ***6.528*** **)**	**1,779 (** ***8.230*** **)**	Calculated

SSA-Sub Saharan and Southern Africa, SEA-South and South-East Asia.

1 The burden of severe malaria in SSA was calculated by applying expert opinion estimates of the percentage of total incidence in SSA that was severe to the entire malaria incidence. In all other regions, expert opinion estimates of the percentage of falciparum malaria incidence that was severe were applied.

^2^Malaria rates presented for total population. It should be noted that 55% of the population in SEA is not at risk of malaria.

In the model, the case fatality rate for severe malaria patients who do not reach a health care facility with the capacity to deliver parenteral treatment was set to 50% (for patients five years and older) and 35% (for under-fives), based on expert opinion in a Tanzanian setting [23]. The case fatality rate for severe bacterial disease patients who do not access such care was set to 16% (all ages), based on an expert opinion [24] informed by a study in children with severe LRI [18], [24], [25]. Patients with severe malaria or bacterial disease who reach an appropriate health care facility were assumed to receive first-line treatment as appropriate, following clinical diagnosis. First-line treatment for severe malaria in SSA is still predominantly parenteral quinine, although this is likely to change soon to artesunate [26]; in SEA artemether is also widely used [10]. Treatment failure rates were set at 26% (five years and over) and 6% (under-five), based on a study of parenteral treatment for severe malaria in hospitalised Asian patients [27], [28]. First-line treatment for severe bacterial disease was assumed to be benzylpenicillin as recommended by WHO [9], [20]. First-line treatment failure rates were set at 9.8% (five years and over) and 5.8% (under-fives) [29], [30]. For both diseases, treatment failure rates were adjusted for patients who accessed healthcare within 6 hours of, and more than 6 hours after rectal intervention administration, using the ratio of respective mortality rates reported in Gomes *et al*. [2]. Following first-line treatment, patients either improve and undertake oral therapy, or deteriorate and die. Some patients with severe malaria recover but with permanent neurological sequelae and the incidence rate for such sequelae was set to 5% [2], [26], [31], [32].

Full coverage of the rectal interventions within relevant target populations was assumed in order to generate estimates of the potential maximum effect. A proportion of the severe febrile patients who receive the rectal formulation will have a disease that is neither malaria nor the target bacterial disease (a ‘spillout’ population). Disease management was not modelled for these patients (as no effects of the rectal formulation are expected), but the decision model captures the cost of the rectal intervention. The percentage of severe febrile patients treated with a rectal antimalarial who do not have malaria was set at 26%, based on data from Gomes *et al*. [2]. The percentage of severe febrile patients treated with a rectal antibacterial who do not have the target bacterial disease, and the percentage of severe febrile patients treated with a combined rectal formulation who have neither severe malaria nor target bacterial disease, were both assumed to be 10%.

In Gomes *et al*. rectal antimalarial treatment reduced mortality by 51% in malaria patients who were alive but not in hospital within 6 hours of treatment administration [2]. Gomes *et al*. reported no effect in participants who present at hospital prior to six hours: our model applies this finding. A 20% reduction in the case fatality rate of malaria was applied in those who did not attend hospital at all. Although Gomes *et al*. reported a reduction of 85% in long-term neurological sequelae [2], no effect was reported in two other studies [26], [33], hence a mid-point value of 42% was applied. Rectal antibacterial treatment was assumed to reduce mortality in severe target bacterial disease patients who are alive but not in hospital within 6 hours of treatment administration by 20%, and in such patients who did not attend hospital by 10%; no effect in such patients reaching appropriate healthcare within six hours was modelled. These values were assumptions based on estimates of parenteral antibiotic treatment effects adjusted downwards to reflect uncertainty concerning adequate rectal absorption. The effect of the combined rectal formulation was modelled by adding the treatment effects of both individual interventions.

All costs were calculated in US dollars for 2005, adjusted for inflation [34]. End user costs of $2.00 and $2.50, comparable with the cost of parenteral antimalarial treatment for under-fives, were used for the antimalarial or antibacterial rectal formulations, and the combined rectal formulation, respectively. The perspective of a healthcare provider was taken, hence the analysis was limited to direct treatment related costs and policy delivery costs. Drug costs reflected a full treatment course and were based on WHO and Kenyan price lists [35], [36]. The cost of oral antibiotics was based on the use of amoxicillin [9], [37]. Dosages reflected WHO treatment guidelines [9], [20]. The cost of a rapid diagnostic test is included in the model as a proxy cost for any diagnostic or co-treatment in addition to appropriate first line therapy [35]. However treatment decisions were not affected by the use of diagnostics. Hospitalisation costs were evaluated using data on cost per day in hospital [38] and duration of hospital admission [14], [39]. The likely cost of deploying rectal treatments was informed by three studies evaluating interventions in communities in developing countries [40], [41], [42]. These studies estimated delivery costs to be $0.03, $0.01, and $0.09 per capita, respectively. A value of $0.02 per capita was used in the base case analysis to reflect all intervention delivery costs (i.e. recruitment and training of providers and education of population) except for drug costs. It was also assumed that delivery mechanisms for an antimalarial, antibacterial and the combined formulations would be similar and therefore their delivery costs per capita would not differ.

Disability Adjusted Life Years (DALYs) were calculated using standard methods [43] without age weighting. Region-specific life tables were used to estimate life expectancy conditional on survival [44] with future life years discounted at 3%. A disability weight of 0.471 was applied for malaria patients surviving with neurological sequelae [45].

Additional costs per death and DALY averted are presented for populations at risk of malaria in SSA and SEA, separately for (1) rectal antimalarial treatment compared with usual practice; (2) rectal antibacterial treatment compared with usual practice; (3) combined antimalarial/antibacterial rectal formulation compared with usual practice; (4) combined antimalarial/antibacterial rectal formulation compared with rectal antimalarial, and (5) combined antimalarial/antibacterial rectal formulation compared with rectal antibacterial.

#### Sensitivity analyses

One-way sensitivity analyses were conducted for key parameters across all comparisons including: hospitalisation and rectal treatment costs; disease incidence rates; neurological sequelae incidence rates; untreated case fatality rates; the treatment effect of the rectal formulations on mortality and long-term disability; and the life tables used within the DALY calculations (**Table 1**).

Variations in access to care were also considered. Access rates from Gomes *et al*. [2] were firstly applied both in usual care and following rectal treatment, and then applied only after rectal treatment. A scenario of lower access rates, both in usual care and following rectal treatment was also considered.

The combined impact of variations in delivery costs and coverage was also evaluated. For delivery costs, a range between zero and $0.10 was considered [40], [41], [42], given the uncertainty over the delivery mode and likely variations in different settings. Coverage levels may also vary, dependent on how interventions are implemented, hence coverage was varied between 100% and 50%.

Further details concerning alternative parameter values and data sources are available in the supplementary material (**Text S1**).

As this study was based on published information and involved no individual participants' data, ethics approval was not required.

## Results

### Burden of disease

#### Malaria

Worldwide incidence of falciparum malaria was estimated at 247 million cases in 2006 (**Table 2**), 241 million of which occurred in SSA and SEA [10]. Falciparum malaria represents 88% of all malaria worldwide: 94% in SSA and 57% in SEA [1]. The total number of severe malaria cases was estimated at 7.2 million in 2006: 6.9 million in SSA and 0.3 million in SEA. Global mortality was estimated to be 881,000 in 2006 [10]: 837,000 deaths occurred in SSA, 736,000 of those in under-fives. Of 40,000 malaria deaths in SEA, 26,000 were in those aged five years and over.

#### Target bacterial disease

The annual global incidence of LRI was estimated at 447 million cases, with 156 million pneumonia cases in under-fives [1], [19] (**Table 2**). There were around three times as many episodes per person per year overall in SSA as compared to SEA. The pattern of severe disease followed that of overall incidence, with 11 million cases in SSA and 17 million in SEA [1], [19]. Annual global mortality from LRI was estimated at 4.2 million deaths, with 1.5 million in SSA (including 1 million pneumonia deaths in under-fives) and 1.7 million in SEA (including 0.6 million pneumonia deaths in under-fives) [1], [21].

#### The combined burden of malaria and target bacterial disease

The combined burden of severe malaria and target bacterial disease was estimated at 46 million cases annually worldwide: 18 million in SSA and 17 million in SEA (**Table 2**). 5 million deaths worldwide were estimated to occur each year across all age groups, 2.3 million of these in SSA and 1.8 million in SEA.

Alternative estimates of the burden of severe febrile illness are summarised in the supplementary material (**Text S1**).

### Cost-effectiveness analysis

The cost-effectiveness results are reported in **Table 3**.

**Table 3:** Cost-effectiveness results.

Comparison	Population	SSA^1^			SEA^1^		
		Additional cost(‘000 US $)	Deaths averted/DALYs averted	Cost per death averted/Cost per DALY averted(US $)	Additional cost(‘000 US $)	Deaths averted/DALYs averted	Cost per death averted/Cost per DALY averted(US $)
**(1) Rectal antimalarial treatment versus usual practice** 2	Under five years	16,990	156,131/4,929,402	109/3	2,556	220/7,427	11,641/344
	Five years and over	18,282	82,297/1,516,042	222/12	27,883	6,654/164,134	4,191/170
	**Total**	**35,272**	**238,428/6,445,443**	**148/** **5**	**30,439**	**6,873/** **171,560**	**4,429/** **177**
**(2) Rectal antibacterial treatment versus usual practice** 2	Under five years	10,708	27,485/753,301	390/14	10,667	10,089/297,756	1,057/36
	Five years and over	35,839	101,778/1,684,796	352/21	36,787	16,435/193,419	2,238/190
	**Total**	**46,547**	**129,263/2,438,097**	**360/** **19**	**47,454**	**26,524/** **491,174**	**1,789/** **97**
**(3) Combined antimalarial and antibacterial rectal formulation versus usual practice** 2	Under five years	27,349	183,616/5,682,703	149/5	12,671	10,308/305,183	1,229/42
	Five years and over	46,054	184,075/3,200,837	250/14	39,889	23,089/357,552	1,728/112
	**Total**	**73,403**	**367,691/8,883,540**	**200/** **8**	**52,560**	**33,397/** **662,735**	**1,574/** **79**
**(4) Combined antimalarial and antibacterial rectal formulation versus rectal antimalarial treatment**	Under five years	10,359	27,485/753,301	377/14	10,115	10,089/297,756	1,003/34
	Five years and over	27,772	101,778/1,684,796	273/16	12,007	16,435/193,419	731/62
	**Total**	**38,131**	**129,263/2,438,097**	**295/** **15**	**22,122**	**26,524/** **491,174**	**834/** **45**
**(5) Combined antimalarial and antibacterial rectal formulation versus rectal antibacterial treatment**	Under five years	16,641	156,131/4,929,402	107/3	2,004	220/7,427	9,127/270
	Five years and over	10,214	82,297/1,516,042	124/7	3,103	6,654/164,134	466/19
	**Total**	**26,856**	**238,428/6,445,443**	**113/** **4**	**5,107**	**6,873/** **171,560**	**743/** **30**

SSA-Sub Saharan and Southern Africa, SEA-South and South-East Asia.

1 These cost-effectiveness results are for the whole region in SSA, and for populations at risk of malaria only in SEA;

2 Usual practice refers to a situation where no rectal treatments for severe febrile illness are widely used.

#### Rectal antimalarial treatment versus usual practice

Compared to usual practice, full coverage with rectal antimalarials would avoid 238,428 deaths in SSA and 6,873 deaths in SEA annually at added healthcare costs of $35 million in SSA and $30 million in SEA. The cost per death avoided is $148 in SSA and $4,429 in SEA, with a cost per DALY averted of $5 in SSA and $177 in SEA.

#### Rectal antibacterial treatment versus usual practice

Compared to usual practice, full coverage with rectal antibacterial treatment would avoid 129,263 deaths in SSA and 26,524 deaths in SEA annually, at added healthcare costs of $47 million in each of SSA and SEA. The cost per death avoided is $360 in SSA and $1,789 in SEA, with a cost per DALY averted of $19 in SSA and $97 in SEA.

#### Combined antimalarial/antibacterial rectal formulation versus usual practice

Compared to usual practice (with rectal antimalarial or antibacterial treatment not in widespread use), a combined rectal formulation would avoid 367,691 deaths in SSA and 33,397 deaths in SEA annually at added healthcare costs of $73 million in SSA and $53 million in SEA. The cost per death avoided is $200 in SSA and $1,574 in SEA, with a cost per DALY averted of $8 in SSA and $79 in SEA.

#### Combined antimalarial/antibacterial rectal formulation versus antimalarial only rectal treatment

Compared to a scenario where rectal antimalarial treatment is already in use, a combined rectal formulation would avoid a further 129,263 deaths in SSA and 26,524 deaths in SEA annually at added healthcare costs of $38 million in SSA and $22 million in SEA. The cost per death avoided is $295 in SSA and $834 in SEA, with a cost per DALY averted of $15 in SSA and $45 in SEA.

#### Combined antimalarial/antibacterial rectal formulation versus antibacterial only rectal treatment

Compared to a scenario where rectal antibacterial treatment is already in use, a combined rectal formulation would avoid a further 238,428 deaths in SSA and 6,873 deaths in SEA at added healthcare costs of $27 million in SSA and $5 million in SEA. The cost per death avoided is $113 in SSA and $743 in SEA, with a cost per DALY averted of $4 in SSA and $30 in SEA.

Cost-effectiveness results for an antibacterial only intervention in populations not at risk of malaria in SEA are presented in the supplementary material (**Text S2**).

### Sensitivity and scenario analyses

**Figure 2:**
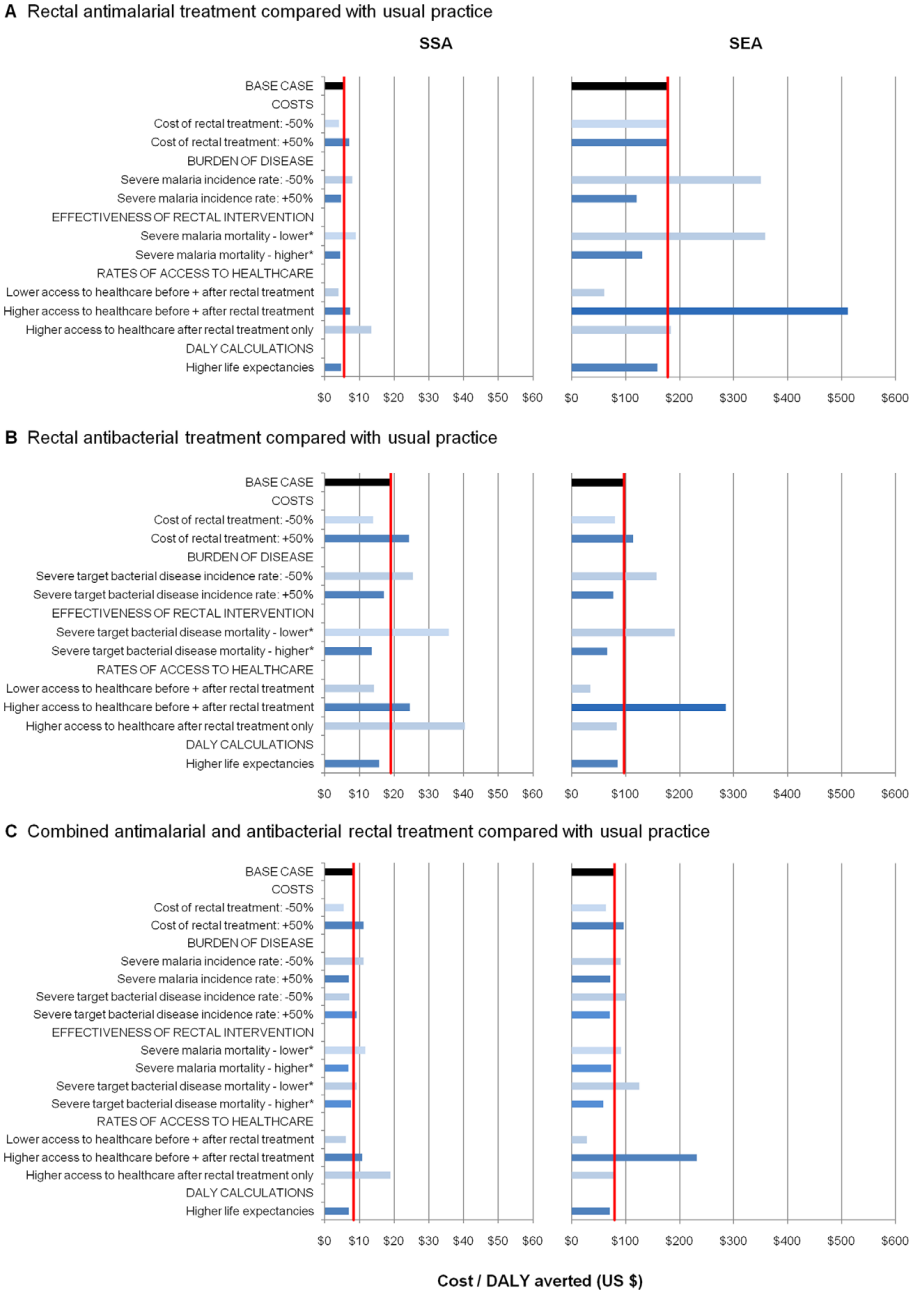
Sensitivity analysis of cost-effectiveness (US$/DALY averted) of rectal formulations for severe febrile illness. Figure 2 presents the impact of changes in values of different parameters on cost-effectiveness results. The three panels present these analyses for rectal antimalarial treatment compared with usual practice (**Panel A**), rectal antibacterial treatment compared with usual practice (**Panel B**), and a combined antimalarial/antibacterial rectal formulation compared with usual practice (**Panel C**). Comparisons between a combined antimalarial/antibacterial rectal formulation and either rectal antimalarial or rectal antibacterial treatment are not presented, however these analyses are reported in **Table S2**. Usual practice refers to a situation where no rectal treatments for severe febrile illness are widely used. Base case estimates of cost per DALY averted are indicated by a red line for each comparison and region. * For full details of parameter variations, see **Table 1** and supplementary **Table S2**.

**Figure 2** summarises the sensitivity analyses for the comparisons of each rectal intervention versus usual practice. Additional details for all parameter variations and comparisons are reported in **Table S2**. All comparisons were moderately sensitive to variations in the cost of the rectal formulation. For the comparison between a combined rectal formulation and usual practice, a 50% reduction in the price of rectal treatment reduced the cost per DALY averted from $8 to $5 in SSA and from $79 to $63 in SEA. Incremental cost-effectiveness estimates were also sensitive to the rectal antibacterial treatment effect. When a smaller treatment effect was applied (10% reduction in mortality in patients alive but not in hospital within 6 hours of rectal treatment administration; 5% reduction in untreated case fatality rate), the cost per DALY averted for the comparison between an antibacterial only intervention and usual practice increased from $19 to $36 in SSA and from $97 to $191 in SEA. Variations in the rectal antimalarial treatment effect had a similar although slightly reduced impact on cost-effectiveness.

The largest changes in incremental cost-effectiveness were observed when healthcare access rates were varied. When higher access rates were applied both before and after rectal treatment introduction in SEA (see **Table 1**) the cost per DALY averted increased from $79 to $232 (combined rectal formulation versus usual practice). Applying higher access rates only after rectal formulation introduction had a notable effect on the comparison between a combined rectal formulation and an antimalarial only rectal formulation in SSA, increasing the cost per DALY averted from $16 to $32: this occurs because more patients benefit from rectal treatment but hospitalisation costs increase substantially. Conversely, when we considered a scenario with lower access rates applied both before and after rectal treatment introduction, the cost per DALY averted decreased for all comparisons in both SSA and SEA.

**Figure 3:**
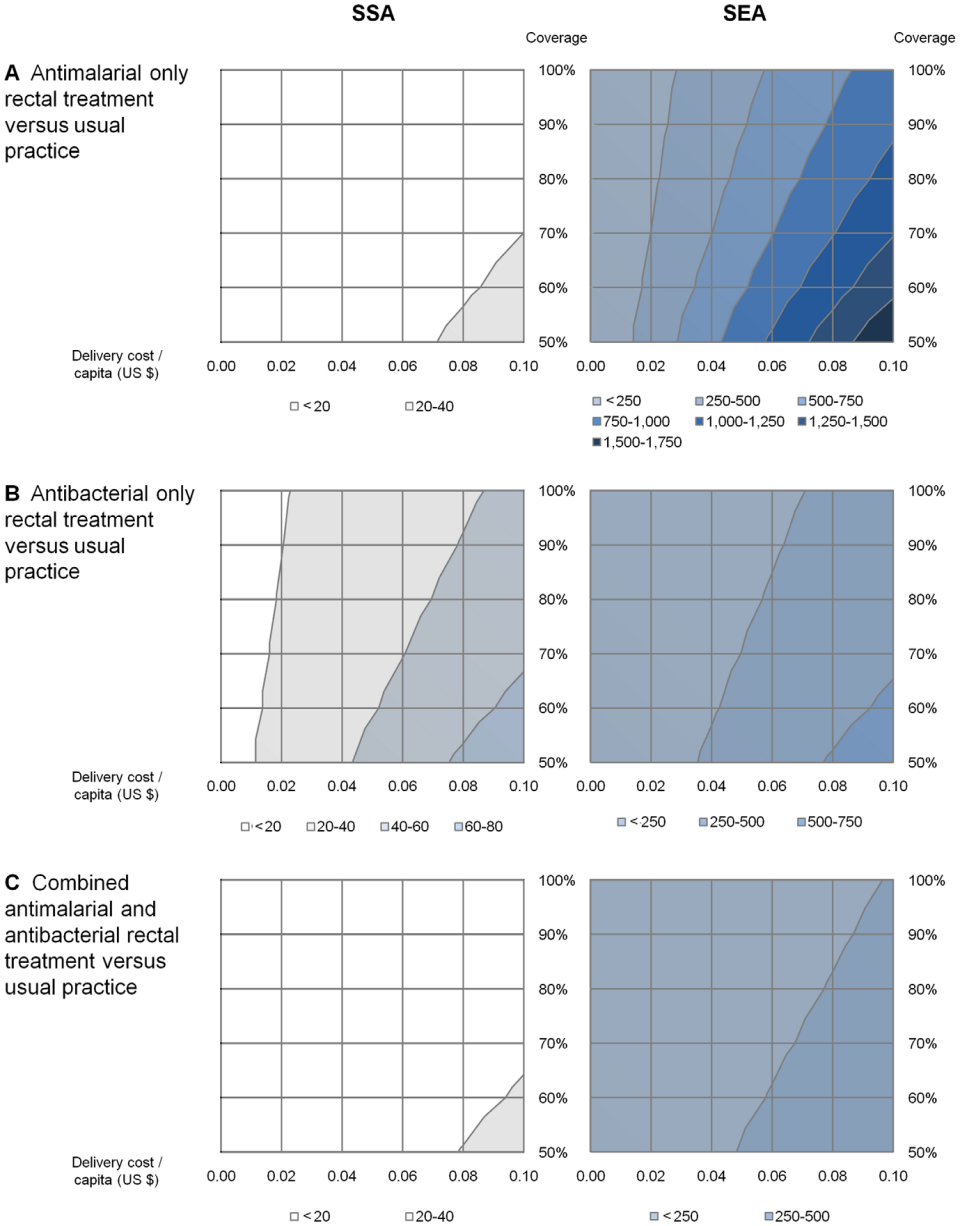
Cost-effectiveness (US$/DALY averted) for different levels of delivery cost and coverage with rectal treatment(s). Figure 3 illustrates how both changes in the cost of deploying rectal treatments (per capita), as well as the coverage levels achieved, could impact on the cost-effectiveness of these interventions. The three panels present these analyses for rectal antimalarial treatment compared with usual practice (**Panel A**), rectal antibacterial treatment compared with usual practice (**Panel B**), and a combined antimalarial/antibacterial rectal formulation compared with usual practice (**Panel C**). The comparisons between a combined rectal formulation and either antimalarial or antibacterial only rectal formulations are not presented, as the delivery costs for the combined rectal formulation and antimalarial or antibacterial only rectal formulations are similar and would difference out without affecting the cost-effectiveness figures presented in **Table 3**. Usual practice refers to a situation where no rectal treatments for severe febrile illness are widely used.

The impact of delivery costs and intervention coverage on the cost-effectiveness results are illustrated in **Figure 3** (**Table S3** provides further detail). In SSA, the cost per DALY averted remained under $100 for all combinations of delivery cost and coverage level considered, for all comparisons. For populations at risk of malaria in SEA there was more variation. With delivery costs of $0.10 per capita and 50% coverage, the cost per DALY averted for the comparison between the combined formulation and current practice increased from $79 to $481.

## Discussion

The total annual burden of severe malaria and severe target bacterial disease is enormous, with 2.3 million related deaths occurring in SSA and 1.8 million in SEA, although considerable uncertainty surrounds mortality in some remote but populous areas within SEA such as North-east India, Myanmar and Indonesia [46], [47], [48], [49]. Emergency pre-referral rectal antimalarial treatment for severe malaria is already available and is incorporated in the WHO treatment guidelines [7], although not yet widely deployed. Antibacterial and combined antimalarial/antibacterial rectal formulations have been proposed to be developed which, if safe, effective and acceptable, could be added to current treatment guidelines. Ensuring rapid, adequate and reliable rectal absorption is a key development objective. This paper uses current disease estimates and costs to suggest that these pre-referral interventions would reduce considerably the burden of severe febrile illnesses in SSA and SEA in a cost-effective manner.

For all comparisons, the incremental cost-effectiveness ratios in SSA were in the range of ‘highly attractive’ interventions ($25 per DALY averted) under World Bank guidance [50], [51], while the ratios in SEA were generally in the range of ‘attractive’ interventions ($150 per DALY averted), under the same guidance. In addition, our results compared favourably with another benchmark for cost-effectiveness, the gross domestic product per capita in the respective countries [52], [53], as well as cost-effectiveness estimates for other interventions for malaria and bacterial infections [54], [55], [56].

This work has a number of potential limitations. First, the burden of disease estimates combine data from several sources; treatment effects and costs were also based on multiple sources from different geographical locations. Ideally, this work would be based on epidemiological and economic data originating from the same population but this has not been possible in our case as two of the intervention studies are not yet developed and no deployment study of antimalarial suppository is yet publically available. Second, whereas treatment effects for antimalarials have been relatively well defined, the effects of antibiotics have not been, and there is considerable uncertainty over both the rate and magnitude of their potentially lifesaving benefit. Third, the categories of access to care used in the decision model were selected to align with the treatment effects of the antimalarial component of the rectal formulation. The time course of illness for patients with severe bacterial infections might however differ, and studies of rectal antibacterial treatment efficacy and effectiveness are needed. Fourth, the effectiveness of rectal treatment may also vary in different population groups due to the presence of underlying conditions. For example, the potential for antibacterial treatment to impact on mortality may be limited in people who develop severe pneumonia alongside existing immunocompromising conditions such as HIV infection [57]. Fifth, the analysis does not consider the impact of these rectal formulations on the emergence or spread of resistance to the active components. Sixth, wider household costs related to seeking treatment and living with long-term sequelae are likely to be significant, particularly if rectal treatments increase the use of health services. Finally, the likely delivery costs, end-user costs and coverage levels of the rectal interventions are unclear. These parameters are likely to depend on intervention implementation and to vary geographically. Further work to study the most cost-effective delivery systems locally is needed.

We have explored the impact of changes in important factors on cost-effectiveness. A reduction in the burden of severe febrile illness due to improved availability of other treatments (for example, as a result of the ACT subsidy scheme, or vaccine development) would reduce the cost-effectiveness of rectal treatments, unless delivery costs were substantially lowered (for example, through improved targeting of interventions). Urbanisation might bring people closer to health facilities, and appropriate interventions at these facilities might reduce the need for rectal treatment. Nonetheless, these separate developments are unlikely to alleviate the need for lifesaving interventions and, as our sensitivity analyses suggest, the interventions remain in the range of cost-effective interventions even under somewhat large changes in the parameters affected.

The cost-effectiveness analyses reported in this paper suggest that rectal formulations of an antibacterial and/or an antimalarial are likely to be cost-effective pre-referral interventions for severe febrile illness in the community. Future work is needed to develop the rectal antibacterial interventions and to study the best ways to make the rectal interventions studied here both available and used in the communities that need them.

## Supporting Information

Table S1 Regional country groupings. Summaries of the burden of severe febrile illness are provided for two regions, Sub-Saharan and Southern Africa (SSA), and South and South East Asia (SEA), alongside worldwide figures. The SSA region contains all African countries excluding those in Northern Africa (Algeria, Egypt, Libya, Morocco and Tunisia), where malaria is not present [World Health Organisation (2008) World Malaria Report]. Five countries were excluded due to a lack of data (Djibouti, Mauritius, Mayotte, Seychelles and Lesotho). The SEA region contains all the countries from two World Health Organisation (WHO) regions: SEARO (South East Asian Regional Office of the WHO) and WPRO (Western Pacific Regional Office of the WHO) which are reported as having malaria present in the 2008 World Malaria Report [World Health Organisation (2008) World Malaria Report].
(0.06 MB DOC)

Table S2 Sensitivity analysis results
(0.35 MB DOC)

Table S3 Scenario analysis results
(0.07 MB DOC)

Text S1 Alternative parameter values and data sources
(0.12 MB DOC)

Text S2 Cost-effectiveness results for an antibacterial only intervention in populations not at risk of malaria in South and South-East Asia
(0.05 MB DOC)
