# Supplementary material for: Cost-Effectiveness of Pre-Referral Antimalarial, Antibacterial, and Combined Rectal Formulations for Severe Febrile Illness
Source: PLoS One. 2010 Dec 29;5(12):e14446. doi: 10.1371/journal.pone.0014446 (PMC3012053; doi:10.1371/journal.pone.0014446)
Supplement: Table S1 — Regional country groupings. Summaries of the burden of severe febrile illness are provided for two regions, Sub-Saharan and Southern Africa (SSA), and South and South East Asia (SEA), alongside worldwide figures. The SSA region contains all African countries excluding those in Northern Africa (Algeria, Egypt, Libya, Morocco and Tunisia), where malaria is not present [World Health Organisation (2008) World Malaria Report]. Five countries were excluded due to a lack of data (Djibouti, Mauritius, Mayotte, Seychelles and Lesotho). The SEA region contains all the countries from two World Health Organisation (WHO) regions: SEARO (South East Asian Regional Office of the WHO) and WPRO (Western Pacific Regional Office of the WHO) which are reported as having malaria present in the 2008 World Malaria Report [World Health Organisation (2008) World Malaria Report]. (0.06 MB DOC) [file pone.0014446.s001.doc]

Cost-Effectiveness of Pre-referral Antimalarial, Antibacterial, and Combined Rectal Formulations for Severe Febrile Illness

James Buchanan, Borislava Mihaylova, Alastair Gray and Nicholas White

**Table S1.** Regional country groupings

| **Sub Saharan and Southern Africa (45)** | | **South and South East Asia (20)** |
| --- | --- | --- |
| Angola | Liberia | Bangladesh |
| Benin | Madagascar | Bhutan |
| Botswana | Malawi | Cambodia |
| Burkina Faso | Mali | China |
| Burundi | Mauritania | India |
| Cameroon | Mozambique | Indonesia |
| Cape Verde | Namibia | Korea, Democratic People's Republic of |
| Central African Republic | Niger | Korea, Republic of |
| Chad | Nigeria | Lao |
| Comoros | Rwanda | Malaysia |
| Congo, Democratic Republic of | Sao Tome and Principe | Myanmar |
| Congo, Republic of | Senegal | Nepal |
| Cote d'Ivoire | Sierra Leone | Papua New Guinea |
| Djibouti | Somalia | Philippines |
| Eritrea | South Africa | Solomon Islands |
| Ethiopia | Sudan | Sri Lanka |
| Equatorial Guinea | Swaziland | Thailand |
| Gabon | Tanzania | Timor-Leste, Democratic Republic of |
| Gambia | Togo | Viet Nam |
| Ghana | Uganda | Vanuatu |
| Guinea | Zambia |  |
| Guinea-Bissau | Zimbabwe |  |
| Kenya |  |  |
