# Supplementary material for: Cost-Effectiveness of Pre-Referral Antimalarial, Antibacterial, and Combined Rectal Formulations for Severe Febrile Illness
Source: PLoS One. 2010 Dec 29;5(12):e14446. doi: 10.1371/journal.pone.0014446 (PMC3012053; doi:10.1371/journal.pone.0014446)
Supplement: Table S2 — Sensitivity analysis results (0.35 MB DOC) [file pone.0014446.s002.doc]

Cost-Effectiveness of Pre-referral Antimalarial, Antibacterial, and Combined Rectal Formulations for Severe Febrile Illness

James Buchanan, Borislava Mihaylova, Alastair Gray and Nicholas White

**Table S2(i)**. Sensitivity analysis - antimalarial only rectal treatment versus usual practice

| **Parameter** | **Change** | **Additional cost (US $)** | **Deaths averted** | **DALYs averted** | **Cost per death averted (US $)** | **Cost per DALY averted (US $)** |
| --- | --- | --- | --- | --- | --- | --- |
| **BASE CASE - SSA^1^** | **-** | **35,271,939** | **238,428** | **6,445,443** | **148** | **5** |
| Rectal treatment cost | 50% decrease | 25,950,621 | 238,428 | 6,445,443 | 109 | 4 |
|  | 50% increase | 44,593,257 | 238,428 | 6,445,443 | 187 | 7 |
| Hospitalisation cost | 50% decrease | 34,669,482 | 238,428 | 6,445,443 | 145 | 5 |
|  | 50% increase | 35,874,397 | 238,428 | 6,445,443 | 150 | 6 |
| Sequelae incidence -severe malaria | 50% decrease | 39,316,798 | 238,428 | 6,087,569 | 165 | 6 |
|  | 50% increase | 31,227,080 | 238,428 | 6,803,317 | 131 | 5 |
| Incidence rate - severe malaria | 50% decrease | 25,266,674 | 119,214 | 3,222,722 | 212 | 8 |
|  | 50% increase | 45,277,204 | 357,642 | 9,668,165 | 127 | 5 |
| Untreated case fatality rate - severe malaria | Low (15% in under fives; 25% in five years and over) | 35,271,939 | 193,074 | 5,299,938 | 183 | 7 |
|  | High (45% in under fives; 75% in five years and over) | 35,271,939 | 283,782 | 7,590,948 | 124 | 5 |
| Treatment failure rate - severe malaria | 50% decrease | 30,543,133 | 164,568 | 4,726,100 | 186 | 6 |
| Rectal treatment effect – severe malaria mortality | Smaller effect | 30,109,017 | 112,433 | 3,422,756 | 268 | 9 |
|  | Larger effect | 38,511,672 | 334,384 | 8,768,880 | 115 | 4 |
| Rectal treatment effect – severe malaria sequelae reduction | No reduction | 43,361,658 | 238,428 | 5,729,695 | 182 | 8 |
|  | 85% reduction | 27,182,221 | 238,428 | 7,161,191 | 114 | 4 |
| Access to healthcare  (percent of cases that reach hospital or die within 6 hours / reach hospital after 6 hours / never reach hospital) | Lower access rates applied (15% / 55% / 30%) | 35,784,900 | 339,177 | 9,148,861 | 106 | 4 |
|  | Higher access rates applied (55% / 32% / 13%) | 34,999,577 | 177,445 | 4,820,877 | 197 | 7 |
|  | Higher access rates applied after rectal treatment only | 107,976,445 | 323,926 | 8,148,781 | 335 | 13 |
| Higher life expectancies | Based on Japanese life tables | 35,271,939 | 238,428 | 7,551,528 | 148 | 5 |
| **BASE CASE - SEA^1^** | **-** | **30,438,797** | **6,873** | **171,560** | **4,429** | **177** |
| Rectal treatment cost | 50% decrease | 30,126,415 | 6,873 | 171,560 | 4,334 | 176 |
|  | 50% increase | 30,751,179 | 6,873 | 171,560 | 4,474 | 179 |
| Hospitalisation cost | 50% decrease | 30,328,383 | 6,873 | 171,560 | 4,413 | 177 |
|  | 50% increase | 30,549,211 | 6,873 | 171,560 | 4,445 | 178 |
| Sequelae incidence - severe malaria | 50% decrease | 30,491,472 | 6,873 | 167,349 | 4,436 | 182 |
|  | 50% increase | 30,386,122 | 6,873 | 175,772 | 4,421 | 173 |
| Incidence rate - severe malaria | 50% decrease | 30,012,538 | 3,437 | 85,780 | 8,733 | 350 |
|  | 50% increase | 30,865,056 | 10,310 | 257,341 | 2,994 | 120 |
| Untreated case fatality rate - severe malaria | Low (15% in under fives; 25% in five years and over) | 30,438,797 | 5,965 | 149,868 | 5,103 | 203 |
|  | High (45% in under fives; 75% in five years and over) | 30,438,797 | 7,781 | 193,253 | 3,912 | 158 |
| Treatment failure rate - severe malaria | 50% decrease | 30,272,245 | 4,345 | 111,684 | 6,968 | 271 |
| Rectal treatment effect – severe malaria mortality | Smaller effect | 30,256,955 | 3,204 | 84,495 | 9,442 | 358 |
|  | Larger effect | 30,552,903 | 9,514 | 234,274 | 3,212 | 130 |
| Rectal treatment effect – severe malaria sequelae reduction | No reduction | 30,544,146 | 6,873 | 163,137 | 4,444 | 187 |
|  | 85% reduction | 30,333,448 | 6,873 | 179,984 | 4,413 | 169 |
| Access to healthcare  (percent of cases that reach hospital or die within 6 hours / reach hospital after 6 hours / never reach hospital) | Lower access rates applied (40% / 40% / 20%) | 30,911,826 | 20,749 | 518,343 | 1,490 | 60 |
|  | Higher access rates applied (93% / 5% / 2%) | 30,297,152 | 2,361 | 59,200 | 12,830 | 512 |
|  | Higher access rates applied after rectal treatment only | 32,340,834 | 7,504 | 176,717 | 4,321 | 183 |
| Higher life expectancies | Based on Japanese life tables | 30,438,797 | 6,873 | 191,728 | 4,429 | 159 |

^1^ SSA-Sub Saharan and Southern Africa, SEA-South and South-East Asia

**Table S2(ii)**. Sensitivity analysis - antibacterial only rectal treatment versus usual practice

| **Parameter** | **Change** | **Additional cost (US $)** | **Deaths averted** | **DALYs averted** | **Cost per death averted (US $)** | **Cost per DALY averted (US $)** |
| --- | --- | --- | --- | --- | --- | --- |
| **BASE CASE - SSA^1^** | **-** | **46,547,086** | **129,263** | **2,438,097** | **360** | **19** |
| Rectal treatment cost | 50% decrease | 33,854,320 | 129,263 | 2,438,097 | 262 | 14 |
|  | 50% increase | 59,239,852 | 129,263 | 2,438,097 | 458 | 24 |
| Hospitalisation cost | 50% decrease | 43,630,493 | 129,263 | 2,438,097 | 338 | 18 |
|  | 50% increase | 49,463,679 | 129,263 | 2,438,097 | 383 | 20 |
| Incidence rate - severe target bacterial disease | 50% decrease | 30,904,248 | 64,631 | 1,219,048 | 478 | 25 |
|  | 50% increase | 62,189,924 | 193,894 | 3,657,145 | 321 | 17 |
| Untreated case fatality rate - severe target bacterial disease | Low (8% in all ages) | 46,547,086 | 110,985 | 2,080,265 | 419 | 22 |
|  | High (24% in all ages) | 46,547,086 | 147,540 | 2,795,929 | 315 | 17 |
| Rectal treatment effect – severe target bacterial disease mortality | Smaller effect | 43,597,014 | 64,631 | 1,214,048 | 675 | 36 |
|  | Larger effect | 49,497,158 | 193,894 | 3,657,145 | 255 | 14 |
| Access to healthcare  (percent of cases that reach hospital or die within 6 hours / reach hospital after 6 hours / never reach hospital) | Lower access rates applied (15% / 55% / 30%) | 48,759,640 | 182,306 | 3,441,841 | 267 | 14 |
|  | Higher access rates applied (55% / 32% / 13%) | 45,372,305 | 98,081 | 1,846,059 | 463 | 25 |
|  | Higher access rates applied after rectal treatment only | 141,377,595 | 196,139 | 3,500,976 | 721 | 40 |
| Higher life expectancies | Based on Japanese life tables | 46,547,086 | 129,263 | 2,971,463 | 360 | 16 |
| **BASE CASE - SEA^1^** | **-** | **47,453,531** | **26,524** | **491,174** | **1,789** | **97** |
| Rectal treatment cost | 50% decrease | 39,109,766 | 26,524 | 491,174 | 1,475 | 80 |
|  | 50% increase | 55,797,295 | 26,524 | 491,174 | 2,104 | 114 |
| Hospitalisation cost | 50% decrease | 46,869,352 | 26,524 | 491,174 | 1,767 | 95 |
|  | 50% increase | 48,037,710 | 26,524 | 491,174 | 1,811 | 98 |
| Incidence rate - severe target bacterial disease | 50% decrease | 38,519,904 | 13,262 | 245,587 | 2,905 | 157 |
|  | 50% increase | 56,387,157 | 39,785 | 736,761 | 1,417 | 77 |
| Untreated case fatality rate - severe target bacterial disease | Low (8% in all ages) | 47,453,531 | 22,318 | 406,954 | 2,126 | 117 |
|  | High (24% in all ages) | 47,453,531 | 30,729 | 575,394 | 1,544 | 82 |
| Rectal treatment effect – severe target bacterial disease mortality | Smaller effect | 46,863,669 | 13,262 | 245,587 | 3,534 | 191 |
|  | Larger effect | 48,043,393 | 39,785 | 736,761 | 1,208 | 65 |
| Access to healthcare  (percent of cases that reach hospital or die within 6 hours / reach hospital after 6 hours / never reach hospital) | Lower access rates applied (40% / 40% / 20%) | 49,903,726 | 79,763 | 1,474,286 | 626 | 34 |
|  | Higher access rates applied (93% / 5% / 2%) | 46,719,836 | 8,930 | 163,714 | 5,232 | 285 |
|  | Higher access rates applied after rectal treatment only | 93,196,989 | 56,477 | 1,119,193 | 1,650 | 83 |
| Higher life expectancies | Based on Japanese life tables | 47,453,531 | 26,524 | 560,386 | 1,789 | 95 |

^1^ SSA-Sub Saharan and Southern Africa, SEA-South and South-East Asia

**Table S2(iii)**. Sensitivity analysis – combined antimalarial/antibacterial rectal treatment versus usual practice

| **Parameter** | **Change** | **Additional cost (US $)** | **Deaths averted** | **DALYs averted** | **Cost per death averted (US $)** | **Cost per DALY averted (US $)** |
| --- | --- | --- | --- | --- | --- | --- |
| **BASE CASE - SSA^1^** | **-** | **73,402,619** | **367,691** | **8,883,540** | **200** | **8** |
| Rectal treatment cost | 50% decrease | 47,966,033 | 367,691 | 8,883,540 | 130 | 5 |
|  | 50% increase | 98,839,205 | 367,691 | 8,883,540 | 269 | 11 |
| Hospitalisation cost | 50% decrease | 69,883,568 | 367,691 | 8,883,540 | 190 | 8 |
|  | 50% increase | 76,921,670 | 367,691 | 8,883,540 | 209 | 9 |
| Sequelae incidence -severe malaria | 50% decrease | 77,447,478 | 367,691 | 8,525,666 | 211 | 9 |
|  | 50% increase | 69,357,760 | 367,691 | 9,241,414 | 189 | 8 |
| Incidence rate - severe malaria | 50% decrease | 63,148,044 | 248,477 | 5,660,819 | 254 | 11 |
|  | 50% increase | 83,657,194 | 486,905 | 12,106,262 | 172 | 7 |
| Incidence rate - severe target bacterial disease | 50% decrease | 54,586,589 | 303,059 | 7,664,492 | 180 | 7 |
|  | 50% increase | 92,218,649 | 432,322 | 10,102,589 | 213 | 9 |
| Untreated case fatality rate - severe malaria | Low (15% in under fives; 25% in five years and over) | 73,402,619 | 322,337 | 7,738,035 | 228 | 9 |
|  | High (45% in under fives; 75% in five years and over) | 73,402,619 | 413,044 | 10,029,045 | 178 | 7 |
| Untreated case fatality rate - severe target bacterial disease | Low (8% in all ages) | 73,402,619 | 349,413 | 8,525,708 | 210 | 9 |
|  | High (24% in all ages) | 73,402,619 | 385,968 | 9,241,372 | 190 | 8 |
| Treatment failure rate - severe malaria | 50% decrease | 68,673,813 | 293,830 | 7,164,197 | 234 | 10 |
| Rectal treatment effect – severe malaria mortality | Reduced effect | 68,239,697 | 241,696 | 5,860,853 | 282 | 12 |
|  | Improved effect | 76,642,352 | 463,647 | 11,206,977 | 165 | 7 |
| Rectal treatment effect – severe malaria sequelae reduction | No reduction | 81,492,337 | 367,691 | 8,167,792 | 222 | 10 |
|  | 85% reduction | 65,312,901 | 367,691 | 9,599,288 | 178 | 7 |
| Rectal treatment effect – severe target bacterial disease mortality | Smaller effect | 70,452,547 | 303,059 | 7,664,492 | 232 | 9 |
|  | Larger effect | 76,352,691 | 432,322 | 10,102,589 | 177 | 8 |
| Access to healthcare  (percent of cases that reach hospital or die within 6 hours / reach hospital after 6 hours / never reach hospital) | Lower access rates applied (15% / 55% / 30%) | 76,128,133 | 521,483 | 12,590,702 | 146 | 6 |
|  | Higher access rates applied (55% / 32% / 13%) | 71,955,476 | 275,526 | 6,666,936 | 261 | 11 |
|  | Higher access rates applied after rectal treatment only | 219,774,486 | 487,305 | 11,649,757 | 451 | 19 |
| Higher life expectancies | Based on Japanese life tables | 73,402,619 | 367,691 | 10,522,991 | 200 | 7 |
| **BASE CASE - SEA^1^** | **-** | **52,560,408** | **33,397** | **662,735** | **1,574** | **79** |
| Rectal treatment cost | 50% decrease | 41,777,083 | 33,397 | 662,735 | 1,251 | 63 |
|  | 50% increase | 63,343,734 | 33,397 | 662,735 | 1,897 | 96 |
| Hospitalisation cost | 50% decrease | 51,865,815 | 33,397 | 662,735 | 1,553 | 78 |
|  | 50% increase | 53,255,002 | 33,397 | 662,735 | 1,595 | 80 |
| Sequelae incidence -severe malaria | 50% decrease | 52,613,083 | 33,397 | 658,523 | 1,575 | 80 |
|  | 50% increase | 52,507,734 | 33,397 | 666,947 | 1,572 | 79 |
| Incidence rate - severe malaria | 50% decrease | 52,092,911 | 29,960 | 576,955 | 1,739 | 90 |
|  | 50% increase | 53,027,906 | 36,833 | 748,515 | 1,440 | 71 |
| Incidence rate - severe target bacterial disease | 50% decrease | 41,540,841 | 20,135 | 417,148 | 2,063 | 100 |
|  | 50% increase | 63,579,976 | 46,659 | 908,322 | 1,363 | 70 |
| Untreated case fatality rate - severe malaria | Low (15% in under fives; 25% in five years and over) | 52,560,408 | 32,489 | 641,042 | 1,618 | 82 |
|  | High (45% in under fives; 75% in five years and over) | 52,560,408 | 34,305 | 684,427 | 1,532 | 77 |
| Untreated case fatality rate - severe target bacterial disease | Low (8% in all ages) | 52,560,408 | 29,191 | 578,515 | 1,801 | 91 |
|  | High (24% in all ages) | 52,560,408 | 37,602 | 746,955 | 1,398 | 70 |
| Treatment failure rate - severe malaria | 50% decrease | 52,393,856 | 30,868 | 602,859 | 1,697 | 87 |
| Rectal treatment effect – severe malaria mortality | Reduced effect | 52,378,567 | 29,728 | 575,670 | 1,762 | 91 |
|  | Improved effect | 52,674,514 | 36,037 | 725,448 | 1,462 | 73 |
| Rectal treatment effect – severe malaria sequelae reduction | No reduction | 52,665,758 | 33,397 | 654,311 | 1,577 | 80 |
|  | 85% reduction | 52,455,059 | 33,397 | 671,158 | 1,571 | 78 |
| Rectal treatment effect – severe target bacterial disease mortality | Smaller effect | 51,970,547 | 20,135 | 417,148 | 2,581 | 125 |
|  | Larger effect | 53,150,270 | 46,659 | 908,322 | 1,139 | 59 |
| Access to healthcare  (percent of cases that reach hospital or die within 6 hours / reach hospital after 6 hours / never reach hospital) | Lower access rates applied (40% / 40% / 20%) | 55,483,633 | 100,511 | 1,992,629 | 552 | 28 |
|  | Higher access rates applied (93% / 5% / 2%) | 51,685,068 | 11,292 | 222,914 | 4,577 | 232 |
|  | Higher access rates applied after rectal treatment only | 99,314,139 | 61,972 | 1,295,910 | 1,603 | 77 |
| Higher life expectancies | Based on Japanese life tables | 52,560,408 | 33,397 | 752,114 | 1,574 | 70 |

^1^ SSA-Sub Saharan and Southern Africa, SEA-South and South-East Asia

**Table S2(iv)**. Sensitivity analysis – combined antimalarial/antibacterial rectal treatment versus antimalarial only rectal treatment

| **Parameter** | **Change** | **Additional cost (US $)** | **Deaths averted** | **DALYs averted** | **Cost per death averted (US $)** | **Cost per DALY averted (US $)** |
| --- | --- | --- | --- | --- | --- | --- |
| **BASE CASE - SSA^1^** | **-** | **38,130,680** | **129,263** | **2,438,097** | **295** | **16** |
| Rectal treatment cost | 50% decrease | 22,015,412 | 129,263 | 2,438,097 | 170 | 9 |
|  | 50% increase | 54,245,947 | 129,263 | 2,438,097 | 420 | 22 |
| Hospitalisation cost | 50% decrease | 35,214,086 | 129,263 | 2,438,097 | 272 | 14 |
|  | 50% increase | 41,047,273 | 129,263 | 2,438,097 | 318 | 17 |
| Incidence rate - severe target bacterial disease | 50% decrease | 19,314,650 | 64,631 | 1,219,048 | 299 | 16 |
|  | 50% increase | 56,946,710 | 193,894 | 3,657,145 | 294 | 16 |
| Untreated case fatality rate - severe target bacterial disease | Low (8% in all ages) | 38,130,680 | 110,985 | 2,080,265 | 344 | 18 |
|  | High (24% in all ages) | 38,130,680 | 147,540 | 2,795,929 | 258 | 14 |
| Rectal treatment effect – severe target bacterial disease mortality | Smaller effect | 35,180,608 | 64,631 | 1,219,048 | 544 | 29 |
|  | Larger effect | 41,080,752 | 193,984 | 3,657,145 | 212 | 11 |
| Access to healthcare  (percent of cases that reach hospital or die within 6 hours / reach hospital after 6 hours / never reach hospital) | Lower access rates applied (15% / 55% / 30%) | 40,343,234 | 98,081 | 3,441,841 | 221 | 12 |
|  | Higher access rates applied (55% / 32% / 13%) | 36,995,898 | 163,379 | 1,846,059 | 377 | 20 |
|  | Higher access rates applied after rectal treatment only | 111,798,041 | 129,263 | 3,500,976 | 678 | 32 |
| Higher life expectancies | Based on Japanese life tables | 38,130,680 | 129,263 | 2,971,463 | 295 | 13 |
| **BASE CASE - SEA^1^** | **-** | **22,121,611** | **26,524** | **491,174** | **834** | **45** |
| Rectal treatment cost | 50% decrease | 11,650,668 | 26,524 | 491,174 | 439 | 24 |
|  | 50% increase | 32,592,555 | 26,524 | 491,174 | 1,229 | 66 |
| Hospitalisation cost | 50% decrease | 21,537,432 | 26,524 | 491,174 | 812 | 44 |
|  | 50% increase | 22,705,790 | 26,524 | 491,174 | 856 | 46 |
| Incidence rate - severe target bacterial disease | 50% decrease | 11,102,044 | 13,262 | 245,587 | 837 | 45 |
|  | 50% increase | 33,141,179 | 39,785 | 736,761 | 833 | 45 |
| Untreated case fatality rate - severe target bacterial disease | Low (8% in all ages) | 22,121,611 | 22,318 | 406,954 | 991 | 54 |
|  | High (24% in all ages) | 22,121,611 | 30,729 | 575,394 | 720 | 38 |
| Rectal treatment effect – severe target bacterial disease mortality | Smaller effect | 21,531,750 | 13,262 | 245,587 | 1,624 | 88 |
|  | Larger effect | 22,711,473 | 39,785 | 736,761 | 571 | 31 |
| Access to healthcare  (percent of cases that reach hospital or die within 6 hours / reach hospital after 6 hours / never reach hospital) | Lower access rates applied (40% / 40% / 20%) | 24,571,807 | 79,763 | 1,474,286 | 308 | 17 |
|  | Higher access rates applied (93% / 5% / 2%) | 21,387,917 | 8,930 | 163,714 | 2,395 | 131 |
|  | Higher access rates applied after rectal treatment only | 66,973,305 | 54,468 | 1,119,193 | 1,229 | 60 |
| Higher life expectancies | Based on Japanese life tables | 22,121,611 | 26,524 | 560,386 | 834 | 39 |

^1^ SSA-Sub Saharan and Southern Africa, SEA-South and South-East Asia

**Table S2(v).** Sensitivity analysis – combined antimalarial/antibacterial rectal treatment versus antibacterial only rectal treatment

| **Parameter** | **Change** | **Additional cost (US $)** | **Deaths averted** | **DALYs averted** | **Cost per death averted (US $)** | **Cost per DALY averted (US $)** |
| --- | --- | --- | --- | --- | --- | --- |
| **BASE CASE - SSA^1^** | **-** | **26,855,533** | **238,428** | **6,445,443** | **113** | **4** |
| Rectal treatment cost | 50% decrease | 14,111,714 | 238,428 | 6,445,443 | 59 | 2 |
|  | 50% increase | 39,599,352 | 238,428 | 6,445,443 | 166 | 6 |
| Hospitalisation cost | 50% decrease | 26,253,076 | 238,428 | 6,445,443 | 110 | 4 |
|  | 50% increase | 27,457,990 | 238,428 | 6,445,443 | 115 | 4 |
| Sequelae incidence - severe malaria | 50% decrease | 30,900,392 | 238,428 | 6,087,569 | 130 | 5 |
|  | 50% increase | 37,110,108 | 357,642 | 6,803,317 | 104 | 4 |
| Incidence rate - severe malaria | 50% decrease | 16,600,958 | 119,214 | 3,222,722 | 139 | 5 |
|  | 50% increase | 37,110,108 | 357,642 | 9,668,165 | 104 | 4 |
| Untreated case fatality rate - severe malaria | Low (15% in under fives; 25% in five years and over) | 26,855,533 | 193,074 | 5,299,938 | 139 | 5 |
|  | High (45% in under fives; 75% in five years and over) | 26,855,533 | 283,782 | 7,590,948 | 95 | 4 |
| Treatment failure rate - severe malaria | 50% decrease | 22,126,727 | 164,568 | 4,726,100 | 134 | 5 |
| Rectal treatment effect – severe malaria mortality | Smaller effect | 21,692,611 | 112,433 | 3,422,756 | 193 | 6 |
|  | Larger effect | 30,095,266 | 334,384 | 8,768,880 | 90 | 3 |
| Rectal treatment effect – severe malaria sequelae reduction | No reduction | 34,945,251 | 238,428 | 5,729,695 | 147 | 6 |
|  | 85% reduction | 18,765,815 | 238,428 | 7,161,191 | 79 | 3 |
| Access to healthcare  (percent of cases that reach hospital or die within 6 hours / reach hospital after 6 hours / never reach hospital) | Lower access rates applied (15% / 55% / 30%) | 27,368,493 | 339,177 | 9,148,861 | 81 | 3 |
|  | Higher access rates applied (55% / 32% / 13%) | 26,583,171 | 177,445 | 4,820,877 | 150 | 6 |
|  | Higher access rates applied after rectal treatment only | 78,396,891 | 291,167 | 8,148,781 | 269 | 10 |
| Higher life expectancies | Based on Japanese life tables | 26,855,533 | 238,428 | 7,551,528 | 113 | 4 |
| **BASE CASE - SEA^1^** | **-** | **5,106,878** | **6,873** | **171,560** | **743** | **30** |
| Rectal treatment cost | 50% decrease | 2,667,316 | 6,873 | 171,560 | 388 | 16 |
|  | 50% increase | 7,546,439 | 6,873 | 171,560 | 1,098 | 44 |
| Hospitalisation cost | 50% decrease | 4,996,463 | 6,873 | 171,560 | 727 | 29 |
|  | 50% increase | 5,217,292 | 6,873 | 171,560 | 759 | 30 |
| Sequelae incidence - severe malaria | 50% decrease | 5,159,552 | 6,873 | 167,349 | 751 | 31 |
|  | 50% increase | 5,054,203 | 6,873 | 175,772 | 735 | 29 |
| Incidence rate - severe malaria | 50% decrease | 4,639,380 | 3,437 | 85,780 | 1,350 | 54 |
|  | 50% increase | 5,574,375 | 10,310 | 257,341 | 541 | 22 |
| Untreated case fatality rate - severe malaria | Low (15% in under fives; 25% in five years and over) | 5,106,878 | 6,873 | 171,560 | 743 | 30 |
|  | High (45% in under fives; 75% in five years and over) | 5,106,878 | 6,873 | 171,560 | 743 | 30 |
| Treatment failure rate - severe malaria | 50% decrease | 4,940,326 | 4,345 | 111,684 | 1,137 | 44 |
| Rectal treatment effect – severe malaria mortality | Smaller effect | 4,925,036 | 3,204 | 84,495 | 1,537 | 58 |
|  | Larger effect | 5,220,983 | 9,514 | 234,274 | 549 | 22 |
| Rectal treatment effect – severe malaria sequelae reduction | No reduction | 5,212,227 | 6,873 | 163,137 | 758 | 32 |
|  | 85% reduction | 5,001,528 | 6,873 | 179,984 | 728 | 28 |
| Access to healthcare  (percent of cases that reach hospital or die within 6 hours / reach hospital after 6 hours / never reach hospital) | Lower access rates applied (40% / 40% / 20%) | 5,579,907 | 20,749 | 518,343 | 269 | 11 |
|  | Higher access rates applied (93% / 5% / 2%) | 4,965,232 | 2,361 | 59,200 | 2,103 | 84 |
|  | Higher access rates applied after rectal treatment only | 6,117,150 | 5,495 | 176,717 | 1,113 | 35 |
| Higher life expectancies | Based on Japanese life tables | 5,106,878 | 6,873 | 191,728 | 743 | 27 |

^1^ SSA-Sub Saharan and Southern Africa, SEA-South and South-East Asia
