# Supplementary material for: Cost-Effectiveness of Pre-Referral Antimalarial, Antibacterial, and Combined Rectal Formulations for Severe Febrile Illness
Source: PLoS One. 2010 Dec 29;5(12):e14446. doi: 10.1371/journal.pone.0014446 (PMC3012053; doi:10.1371/journal.pone.0014446)
Supplement: Table S3 — Scenario analysis results (0.07 MB DOC) [file pone.0014446.s003.doc]

Cost-Effectiveness of Pre-referral Antimalarial, Antibacterial, and Combined Rectal Formulations for Severe Febrile Illness

James Buchanan, Borislava Mihaylova, Alastair Gray and Nicholas White

**Table S3**: Scenario analysis: Cost per DALY averted (US $) when delivery costs and levels of coverage are varied in populations at risk of malaria in SSA and SEA

| **Region** | **Delivery cost per capita (US $)** | **Antimalarial only rectal treatment versus usual practice** | | | | | | **Antibacterial only rectal treatment versus usual practice** | | | | | | **Combined antimalarial/antibacterial rectal formulation versus usual practice** | | | | | |
| --- | --- | --- | --- | --- | --- | --- | --- | --- | --- | --- | --- | --- | --- | --- | --- | --- | --- | --- | --- |
|  |  | **Coverage (%)** | | | | | | **Coverage (%)** | | | | | | **Coverage (%)** | | | | | |
|  |  | **100%** | **90%** | **80%** | **70%** | **60%** | **50%** | **100%** | **90%** | **80%** | **70%** | **60%** | **50%** | **100%** | **90%** | **80%** | **70%** | **60%** | **50%** |
| **SSA^1^** | 0.00 | 3 | 3 | 3 | 3 | 3 | 3 | 13 | 13 | 13 | 13 | 13 | 13 | 7 | 7 | 7 | 7 | 7 | 7 |
|  | 0.02 | 5 | 6 | 6 | 6 | 7 | 8 | 19 | 20 | 21 | 22 | 23 | 25 | 8 | 8 | 9 | 9 | 9 | 10 |
|  | 0.04 | 8 | 8 | 9 | 10 | 11 | 13 | 25 | 27 | 28 | 31 | 34 | 38 | 10 | 10 | 11 | 11 | 12 | 13 |
|  | 0.06 | 10 | 11 | 12 | 13 | 15 | 17 | 32 | 34 | 36 | 40 | 44 | 50 | 12 | 12 | 13 | 14 | 15 | 17 |
|  | 0.08 | 13 | 14 | 15 | 17 | 19 | 22 | 38 | 41 | 44 | 49 | 55 | 63 | 13 | 14 | 15 | 16 | 18 | 20 |
|  | 0.10 | 15 | 16 | 18 | 20 | 23 | 27 | 44 | 48 | 52 | 58 | 65 | 75 | 15 | 16 | 17 | 19 | 21 | 24 |
|  |  | **100%** | **90%** | **80%** | **70%** | **60%** | **50%** | **100%** | **90%** | **80%** | **70%** | **60%** | **50%** | **100%** | **90%** | **80%** | **70%** | **60%** | **50%** |
| **SEA**^1^ | 0.00 | 5 | 5 | 5 | 5 | 5 | 5 | 36 | 36 | 36 | 36 | 36 | 36 | 35 | 35 | 35 | 35 | 35 | 35 |
|  | 0.02 | 177 | 197 | 221 | 251 | 292 | 350 | 97 | 103 | 112 | 122 | 137 | 157 | 79 | 84 | 90 | 98 | 109 | 124 |
|  | 0.04 | 350 | 388 | 436 | 498 | 580 | 695 | 157 | 170 | 187 | 208 | 237 | 277 | 124 | 134 | 146 | 162 | 183 | 213 |
|  | 0.06 | 522 | 580 | 652 | 744 | 867 | 1,040 | 217 | 237 | 262 | 295 | 338 | 398 | 169 | 183 | 202 | 226 | 258 | 303 |
|  | 0.08 | 695 | 771 | 867 | 990 | 1,155 | 1,385 | 277 | 304 | 338 | 381 | 438 | 518 | 213 | 233 | 258 | 290 | 332 | 392 |
|  | 0.10 | 867 | 963 | 1,083 | 1,237 | 1,442 | 1,730 | 338 | 371 | 413 | 467 | 538 | 639 | 258 | 283 | 314 | 354 | 407 | 481 |

^1^ The comparison between a combined rectal formulation and an antimalarial or antibacterial only formulation is not considered in either region for this scenario analysis, as the infrastructure required to implement the combined rectal formulation is likely to already be in place in each case. The results underlie the graphs presented in **Figure 3**.
