# Supplementary material for: Cost-Effectiveness of Pre-Referral Antimalarial, Antibacterial, and Combined Rectal Formulations for Severe Febrile Illness
Source: PLoS One. 2010 Dec 29;5(12):e14446. doi: 10.1371/journal.pone.0014446 (PMC3012053; doi:10.1371/journal.pone.0014446)
Supplement: Text S1 — Alternative parameter values and data sources (0.12 MB DOC) [file pone.0014446.s004.doc]

Cost-Effectiveness of Pre-referral Antimalarial, Antibacterial, and Combined Rectal Formulations for Severe Febrile Illness

James Buchanan, Borislava Mihaylova, Alastair Gray and Nicholas White

**Text S1**: Alternative parameter values and data sources

**Cost of rectal treatment**

The costs of rectal treatment applied in the decision model are assumed costs which are comparable with parenteral antimalarial treatment in under fives. In practice, these costs are likely to vary by dosage, and might also be much lower overall if the treatments were in widespread use, with a consequent effect on the cost-effectiveness estimates. Data on the actual cost of antimalarial only rectal treatment is limited. Data for Benin indicate that the price of rectal artesunate at retail pharmacies is around $0.80 for a 50mg dose and $1.30 for a 200mg dose [1]. However these estimates were based on a survey of government sources and private suppliers to estimate drug mark-ups in the private commercial sector distribution chain. Drug mark-ups from manufacturing prices vary considerably both between and within countries and may also differ in public systems [1,2,3]. Furthermore, retail pharmacies may not represent the final point along the distribution chain required to deliver rectal treatment to the target population in the community.

**Access to care and compliance with referral in Sub-Saharan and Southern Africa (SSA), and South and South East Asia (SEA)**

The decision model developed to simulate the usual management of severe febrile illness differentiates between patients who attended a healthcare facility with the capacity to deliver parenteral treatment within 6 hours of administration of rectal treatment, attended such care after 6 hours, or did not attend such care. These access categories reflect the treatment effects reported in Gomes *et al*., a large multicentre trial of rectal artesunate versus placebo conducted in SEA and SSA [4]. The trial reported data on access to healthcare for approximately 12,000 patients of all ages with severe malaria who were referred to hospital, indicating that 93% of patients in Bangladesh (SEA) and 55% of patients in Ghana and Tanzania (SSA) reached hospital (or died) within 6 hours. Furthermore, 5% in SEA and 32% in SSA reached hospital after 6 hours. The remainder never reached hospital.

These access rates may reflect the fact that some study participants were given free transportation to hospital upon referral, some were guaranteed access to hospital care, and treatments were heavily subsidised or free of charge on arrival for others. As access rates observed in the community may differ from those reported in Gomes *et al*., the decision model assumes lower rates, as detailed in **Table 1** [4].

A number of community studies have also attempted to estimate access to care or compliance with referral (which could reflect ease of access, amongst other factors) in alternative settings and scenarios. Overall, a varied picture of access to care and care seeking behaviour is presented. A study undertaken in Uganda presents data on care seeking behaviour for children under five who ultimately died of pneumonia [5]. Of the 44 children included in the study, 10 children (23%) did not reach a qualified healthcare provider before dying. Of those that did reach a healthcare provider, the medium duration from illness recognition to seeking care was 2 days. A further study in Uganda, this time considering the effectiveness of Home Based Management of fever in increasing access to prompt presumptive malaria treatment, also reported data on access to care [6]. Of 77 children under five who presented in the community with malaria related symptoms (not necessarily severe) and who were referred to a health centre, 67 (87%) reached such a health facility. A third study in Sudan considered adherence to referral to hospital by health workers based on IMCI guidelines, again in children under five [7]. 136 children were referred to a higher level health facility with varying degrees of urgency (3% were urgent), and of these, 90 (66%) attended such a facility. Of those that reached hospital, only 53 (59%) arrived the same day.

A fourth study in Tanzania focused specifically on care-seeking patterns for subsequently fatal malaria [8]. The study population was 320 under fives who lived in a rural area with stable malaria transmission. Of those who died from malaria, 21% failed to either seek modern care (defined as forms of healthcare that are not traditional healers) or any care at all. A final SEA study considered referral from first-level health facilities to hospital for children under five with severe pneumonia in Bangladesh, both before and after the full implementation of revised IMCI guidelines [9]. Of 195 children under five referred prior to the introduction of the guidelines, 158 (81%) did not comply with referral. After the guidelines were introduced, 107 were referred and 57 (53%) did not comply.

Alongside these community studies, often conducted in just one country, household surveys have also been conducted by international organisations to collect data on health indicators in multiple countries using comparable data collection techniques. Multiple Indicator Cluster Surveys (MICS) are conducted by UNICEF every five years and collect data across a sample of households in each country to enable the estimation of the percentage of under fives with suspected pneumonia taken to an appropriate health provider, amongst other parameters [10]. Data for 2006 indicate that the percentage of under fives fulfilling this criteria was 40% in SSA and 62% in South Asia. However these data are specific to a population subgroup (under fives) and the definition of pneumonia (and therefore the severity of disease) is unclear.

Demographic and Health Surveys (DHS) are also conducted by the United States Agency for International Development (USAID) approximately once every five years, and these collect similar data to MICS across a sample of households in a country using standardised questionnaires [11]. Both UNICEF and USAID attempt to harmonise the questions asked on the separate surveys to facilitate comparability between studies. The DHS also ask respondents questions to enable the estimation of the percentage of under fives with suspected pneumonia taken to an appropriate health provider. Results for the countries which took part in the Gomes *et al*. study [4] are available for dates ranging from 2003-2007, and all estimates are close to those reported in the MICS.

**Delivery costs**

Several potential modes of delivery exist (e.g. using mothers as carers, or community health workers). The final delivery cost will depend on the delivery mode chosen and different implementation models are likely to be applicable in different settings [12]. A small number of studies have considered the cost of implementing new interventions in the geographical areas and population groups of interest.

One study considered the costs of introducing Artemisinin Combination Therapy (ACT) in Tanzania through public health facilities [13]. Costs considered included those incurred to recruit and train providers, and to educate the population. Excluding drug purchase and distribution, a cost of $0.09 per capita per annum was reported at the national level.

A second community randomised trial considered the introduction of Intermittent Preventive Treatment in Infants (IPTi) across several districts in Tanzania [14]. Both the cost of developing a national delivery system and also the cost of implementing IPTi at the district level were considered. Approximately $1m was required in the first year ($0.03 per capita) to develop and implement the strategy nationally. The running costs for one year were calculated as approximately $0.5m ($0.01 per person). A further Tanzanian study considered the cost of changing national malaria treatment policy from chloroquine to SP [15]. Costs associated with training, guideline preparation and communications were estimated: the intervention cost was excluded. The total incremental cost was estimated to be $0.8m over 3 years ($0.01 per capita per annum).

**Alternative estimates of the burden of severe febrile illness**

Malaria incidence

The World Malaria Report (WMR) estimate of worldwide malaria incidence was 247 million cases in 2006 [16]. The 2009 WMR estimated that incidence in 2008 was 243 million cases [17,18]. Of the alternative data sources, initial estimates from the Malaria Atlas Project (MAP) suggested that the worldwide incidence of Plasmodium falciparum malaria was 515 million episodes in 2002, within a range of 300-660 million episodes [19,20]. The 2008 WHO Global Burden of Disease study estimated global incidence to be 241 million cases in 2004 [17]. Roca-Feltrer *et al* estimated total morbidity in SSA in under fives in the year 2000 to be 116 million cases [21]. WMR 2008 incidence data is reported in this paper as this data was recently estimated (2006), available for the populations of interest, and the data year coincided with that for the other burden of disease data sources reported in the paper.

Severe malaria incidence

The total number of severe malaria cases estimated in the current paper was 7.2 million in 2006 [16,22,23,24,25]. The 2008 WMR reports estimates of the annual number of hospitalised malaria cases in a subset (54) of all malarial countries [16]. These estimates are unlikely to represent all severe malaria cases as those who never attend hospital or die in the community are excluded. However this data could be viewed as a possible lower bound for the actual burden of severe malaria. To generate an estimate of worldwide hospitalised cases from this subset of countries, for countries lacking hospitalisation data, malaria hospitalisations were estimated as a percentage of total incidence by calculating regional averages, then applying these averages to country level incidence. Total hospitalised malaria cases worldwide were estimated to be 4.0 million in 2006, predominantly occurring in SSA and SEA [16]. Although there is some agreement between the estimates reported in this paper and WMR hospitalisation data for SEA, in general the regional figures mask significant disparities between the two data sources at the country level.

Roca-Feltrer *et al* also estimated the burden of severe malaria in under fives, but these estimates were based on reported data for a subset of severe malaria syndromes (including cerebral malaria, severe malarial anaemia, respiratory distress and persistent neurological sequelae resulting from cerebral malaria, but excluding malaria-attributable anaemia and the effects of comorbidity) [21]. They are not therefore directly comparable with the estimates reported in this paper.

A 2007 paper which evaluated the costs of scaling up malaria interventions worldwide also estimated total severe cases globally. Detailed information on the calculations made is not accessible: however incidence rates between 0.005 and 0.04 per person per year were applied in different settings. The total number of severe episodes was estimated to be 14 million in 2005: 10.7 million in Africa, and 3.3 million in Asia and the Americas. Severe cases represented 3-5% of all episodes [26].

Malaria mortality

Global mortality, used in the current paper, was estimated to be 881,000 in 2006 [16]. The WMR estimates of malaria mortality are similar to those reported by the WHO GBD study for 2004 [17]. An earlier study (DCPP) reported higher estimates for under fives of 1.1 million deaths in 2001, although as in the WHO GBD study there was agreement on the five years and over mortality burden [27]. WMR 2008 mortality data is reported, again because the data year coincides with that for other recently released data sources used in this paper, and because data were available for all populations of interest.

Target bacterial disease incidence

The annual global incidence of lower respiratory infections, used in the current paper, was estimated at 447 million cases [17,28]. Two studies have estimated the burden of disease caused by *Streptococcus pneumoniae* and *Haemophilus influenzae* type b in under fives [29,30]. The combined estimate of incidence from these two bacterium in under fives was 21.7 million cases worldwide: 5.7 million in SSA and 12.5 million in SEA. The authors noted that around 8% of clinical pneumonia was likely to be caused by pneumococcus and 5% by Hib, hence these estimates are not directly comparable with those presented in this paper.

Severe target bacterial disease incidence

The annual global incidence of severe target bacterial disease, used in this paper, was estimated to be 38 million cases. This is based on work by Rudan *et al*., who estimated that 8.6% of pneumonia cases in under-fives develop into severe pneumonia (the median value from six community-based longitudinal studies), and use this estimate to calculate the number of severe pneumonia cases in under-fives for all countries in 2006 [31]. We apply the same incidence rate to the five years and over population. No alternative estimates are available.

Target bacterial disease mortality

Annual global mortality, used in the current paper, was estimated at 4.2 million deaths, with 1.5 million in SSA and 1.7 million in SEA [17,32]. The overall 2004 GBD lower respiratory infection mortality estimate is comparable with the 2001 estimate of lower respiratory infection mortality in the Disease Control Priorities in Developing Countries (DCPP) survey (see table below). The breakdown of mortality between age groups above and below the age of five differs depending on the data source. The residual estimate of the mortality burden we currently use for the five years and over age group is approximately midway between the GBD 2004 and the DCPP 2001 estimates; the UNICEF 2004 estimate of pneumonia mortality in under fives is slightly higher than both the estimate of lower respiratory infection mortality in under fives reported in the GBD (2004) and also the DCPP (2001) estimates. Deaths per 1,000 incident cases range between 11.4-13.1 in under fives, and between 6.2-8.2 in the five years and over age group.

| **Mortality, thousands** (*Mortality rate per 1,000 incident cases*) | **Various sources - 2004 data** | **Global Burden of Disease 2008 - 2004 data [17]** | **Disease Control Priorities in Developing Countries 2006 - 2001 data [27]** |
| --- | --- | --- | --- |
| Under five years | 2,044 (*13.129*) [32] | 1,781 (*11.440*) | 1,944 (*12.487*) |
| Five years and over | 2,133 (*7.327*) [Residual] | 2,395 (*8.229*) | 1,809 (*6.214*) |
| Total | 4,177 (*9.348*) [17] | 4,177 (*9.348*) | 3,753 (*8.399*) |

O’Brien *et al* and Watt *et al* also estimate under five mortality resulting from disease caused by *Streptococcus pneumoniae* and *Haemophilus influenzae* type b [29,30]. The two studies combined estimate 1 million such deaths annually worldwide, with 0.5 million in SSA and 0.3 million in SEA, and an overall estimate of deaths rate per 1,000 incident cases of 48 for these pathogens.
