# Supplementary material for: Cost-Effectiveness of Pre-Referral Antimalarial, Antibacterial, and Combined Rectal Formulations for Severe Febrile Illness
Source: PLoS One. 2010 Dec 29;5(12):e14446. doi: 10.1371/journal.pone.0014446 (PMC3012053; doi:10.1371/journal.pone.0014446)
Supplement: Text S2 — Cost-effectiveness results for an antibacterial only intervention in populations not at risk of malaria in South and South-East Asia (0.05 MB DOC) [file pone.0014446.s005.doc]

Cost-Effectiveness of Pre-referral Antimalarial, Antibacterial, and Combined Rectal Formulations for Severe Febrile Illness

James Buchanan, Borislava Mihaylova, Alastair Gray and Nicholas White

**Text S2:** Cost-effectiveness results for an antibacterial only intervention in populations not at risk of malaria in South and South-East Asia (SEA)

The cost-effectiveness results for an antibacterial only intervention in populations at risk of malaria are reported in **Table 3**. We also considered the cost-effectiveness of an antibacterial only intervention in populations not at risk of malaria in SEA.

| **Comparison** | **Population** | **SEA** | | |
| --- | --- | --- | --- | --- |
|  |  | **Additional cost**  **(‘000 US $)** | **Deaths averted /**  **DALYs averted** | **Cost per death averted /**  **Cost per DALY averted**  **(US $)** |
| **Rectal antibacterial treatment versus usual practice^1^** | Under five years | 12,874 | 12,176 /  359,349 | 1,057 /  36 |
|  | Five years and over | 44,396 | 19,835 /  233,429 | 2,238 /  190 |
|  | **Total** | **57,270** | **32,010 /**  **592,779** | **1,789 /**  **97** |

^1^ Usual practice refers to a situation where no rectal treatments for severe febrile illness are widely used

Full coverage with rectal antibacterial treatment in populations not at risk of malaria would add $57 million annually to healthcare costs in SEA, compared to usual practice, avoiding 32,010 deaths. The cost per death avoided is $1,789 and the cost per DALY averted is $97. These cost-effectiveness results are identical to those for the introduction of this intervention in malarious regions of SEA, reflecting the fact that the costs of treatment are a constant marginal value per treated case in our decision model.
